# Unified End-to-End YOLOv5-HR-TCM Framework for Automatic 2D/3D Human Pose Estimation for Real-Time Applications

**DOI:** 10.3390/s22145419

**Published:** 2022-07-20

**Authors:** Hung-Cuong Nguyen, Thi-Hao Nguyen, Rafal Scherer, Van-Hung Le

**Affiliations:** 1 Faculty of Engineering Technology, Hung Vuong University, Viet Tri City 35100, Vietnam; cuongnh@hvu.edu.vn (H.-C.N.); haont@hvu.edu.vn (T.-H.N.); 2 Department of Intelligent Computer Systems, Czestochowa University of Technology, 42-218 Czestochowa, Poland; rafal.scherer@pcz.pl; 3 Faculty of Basic science, Tan Trao University, Tuyen Quang City 22000, Vietnam

**Keywords:** YOLOv5, HR, 2D/3D human pose estimation, Convolutional Neural Network, Temporal Convolution Model, Pose-based Sports Application

## Abstract

Three-dimensional human pose estimation is widely applied in sports, robotics, and healthcare. In the past five years, the number of CNN-based studies for 3D human pose estimation has been numerous and has yielded impressive results. However, studies often focus only on improving the accuracy of the estimation results. In this paper, we propose a fast, unified end-to-end model for estimating 3D human pose, called YOLOv5-HR-TCM (YOLOv5-HRet-Temporal Convolution Model). Our proposed model is based on the 2D to 3D lifting approach for 3D human pose estimation while taking care of each step in the estimation process, such as person detection, 2D human pose estimation, and 3D human pose estimation. The proposed model is a combination of best practices at each stage. Our proposed model is evaluated on the Human 3.6M dataset and compared with other methods at each step. The method achieves high accuracy, not sacrificing processing speed. The estimated time of the whole process is 3.146 FPS on a low-end computer. In particular, we propose a sports scoring application based on the deviation angle between the estimated 3D human posture and the standard (reference) origin. The average deviation angle evaluated on the Human 3.6M dataset (Protocol #1–Pro #1) is 8.2 degrees.

## 1. Introduction

**Figure 1:**
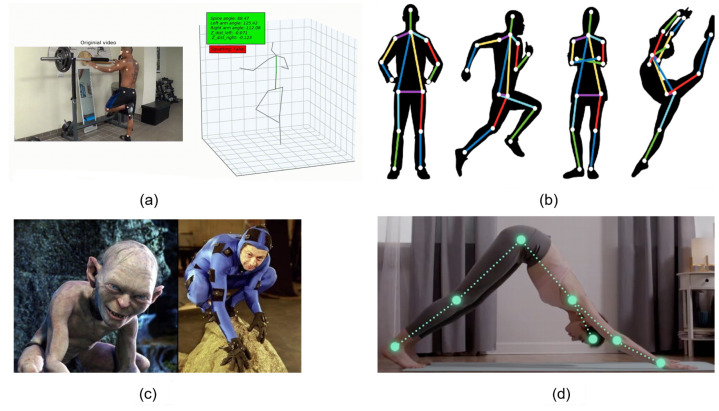
Illustrating of using human posture estimation in weightlifting practice (**a**) [5], healthcare, sports (**b**,**d**) [1], and robotics (**c**) [3].

Human pose estimation is regarded as one of the most interesting research areas in computer vision. It is applied to many fields such as healthcare, sports [1], activity recognition [2], motion capture and augmented reality, training robots, motion tracking for consoles [3], etc. Barla [4] presented seven applications of human pose estimation. In particular, sports have begun to use the results of human pose estimation in practice and competition [5]. Some applications are illustrated in Figure 1.

Human pose estimation is defined as the process of localizing human joints (also known as keypoints—elbows, wrists, etc.) in images or videos. There are two study directions for estimating human pose from images/videos: 2D human pose estimation and 3D human pose estimation. Two-dimensional human pose estimation is an intermediate result for the 3D human pose estimation. Based on the approach of Zhou et al. [6], 3D human pose estimation results are highly dependent on 2D human pose estimation. In the last five years, this task has been gaining much attention, and the research on 3D human pose estimation helps to build intuitive and important applications in robotics, for example training a robot to perform a certain task according to human activity. So far, many kinds of research have focused on improving the accuracy of human pose estimation in the 2D/3D space. Recently, there has been the research by Mehta et al. [7], who proposed an application to estimate 3D human pose in real-time (30 frames per second on a six-core Xeon CPU, 3.8 GHz, and a single Titan X (Pascal architecture) GPU) from RGB images. However, the accuracy of this 3D human pose estimation is not particularly high. To be able to apply the results of 3D human pose estimation in sports analysis, in scoring sports performances, a high-precision system of 3D human pose estimation and time is required. The fast computational time follows the speed of movement and performance of movements and tests in sports. Especially to reduce the processing time, the system is capable of performing 3D human pose estimation based on RGB images, which is common and easily collected data. Especially, the system is initially applied in a gym fitness center or to some non-competitive sports such as weightlifters, weightlifting competitions, single skating, and gymnastics. This means that in the setting, there is only one performer. Therefore, in this paper, we propose a unified end-to-end model for estimating 3D human pose from the RGB image of a monocular camera. The initial data for building the system are the RGB image and 3D human pose annotation. System run and test data include color images only. To obtain a model with high accuracy and fast computation time in 3D human pose estimation, the modeling steps must have high accuracy and a fast processing time: human detection, 2D human pose estimation, and 3D human pose estimation. The steps to build the system are described below.

In this paper, we are interested in both 2D human pose estimation and 3D human pose estimation problems from monocular RGB images or videos. Two deep-learning-based approaches can be used to estimate 2D human poses. The first is the regression method, which applies a deep neural network to learn a mapping from the input image to body joints or parameters of human body models to predict the keypoints on the human (keypoint-based). The second is the body part detection methods to predict the approximate locations of body parts and joints (bodypart-based). Deep learning (DL) networks have achieved remarkable results in estimation tasks. However, they still face many challenges such as heavy occlusion, a partially visible human body, and low image resolution. Sudharshan [8] presented some typical studies [9,10,11,12,13,14,15,16] on estimating 2D human posture in images or videos. In Table 2 of [12], the authors showed the results obtained by the high-resolution network (HR) comparing the above methods for 2D human pose estimation on the COCO [17] dataset. HR is the most accurate across different configurations. Li et al. [18] used HR as a backbone for 2D human pose estimation on cropped human images of the Human 3.6M dataset [19]. As the Human 3.6M dataset contains 548,819 images of Pro #1 for testing, manually marking the data area of the person in the image would take a long time. This difficulty is very dependent on the person conducting the cropping and HR’s estimated data area in the human data region, without regard for other regions in the image. This problem of detecting people in the image is considered to be 100% accurate. However, when applied to real problems, no approach is appropriate.

Three-dimensional human pose estimation is currently of great research interest in the field of computer vision. Recently, there have been many surveys on this issue [20,21,22,23]. According to the surveys, 3D human pose estimation from monocular RGB images or video is based on three methods: direct estimation method, 2D to 3D lifting method, and human mesh recovery method. Current studies on 3D human pose estimation have very impressive results. In the study of Li et al. [18], the average error of the 3D human pose estimation on the Human 3.6M dataset was 49.7 mm (Pro #1) and 37.7 mm (Pro #2). In [24], Chen et al. proposed a study with a mean error on the Human 3.6M dataset of 46.3 mm (Pro #1). However, like many other studies, this study is not interested in the 3D human pose estimation processing time.

**Figure 2:**
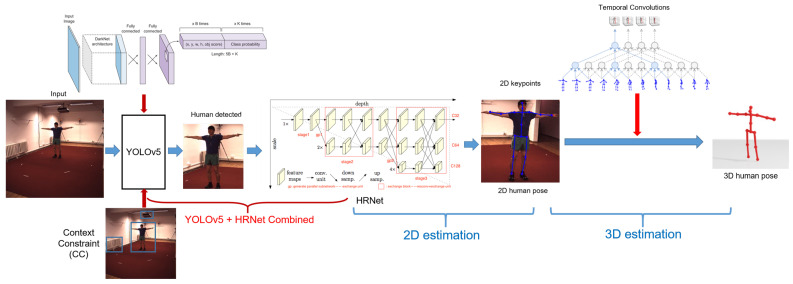
The unified end-to-end YOLOv5-HR-TCM framework for 3D human pose estimation from RGB images taken by a monocular camera.

We propose a unified end-to-end model, called YOLOv5-HR-TCM, for the real-time estimation of 3D human pose, as shown in Figure 2. The model that we propose is fully automatic from end-to-end in estimating 3D human pose from the monocular RGB images or video. The model we propose includes three stages: human detection, 2D human pose estimation, and 3D human pose estimation. In the first stage, we combine the advantages of processing speed and a contextual constraint (CC) into a pre-trained YOLOv5 network [25,26] for detecting a person in a crowd to detect the human in the RGB image. In the second stage, we use a pre-trained model of HR for estimating 2D keypoints/2D human pose in the RGB image. The third stage is the 3D human pose estimation by the temporal convolutions model (TCM) [27]. Unlike previous studies on estimating 3D human pose from a single-camera RGB image, our approach is a combination of CNNs that have the best performance currently in the tasks of person detection, 2D human pose estimation, and 3D human pose estimation. Finally, we apply a simple computational technique to compute the angle between the bone ground-truth and the estimated bone for scoring in a sports application. Our framework is fully automated and executes in real-time on a PC with a low configuration, with takes the input as the monocular RGB images or video and the ground-truth of the 3D human pose. The outputs are the estimated 3D human pose in 3D space and the average deviated angle of the bones.

The main contributions of the paper are as follows:

- We propose a unified end-to-end framework for automatic 3D human pose estimation. The framework is a combination of high-performance CNNs to perform sequential tasks: human detection, 2D human pose estimation, and 3D human pose estimation.
- We embedded efficient contextual constraints (CCs) into YOLOv5 for human detection and HR for 2D keypoint estimation/2D human pose estimation in images or video, called YOLOv5 + CC + HR combined. We also evaluated the results in detail at this stage on the Human 3.6M dataset.
- We applied the TCM and semi-supervised training method in our framework using the 2D human pose estimation results in the fine-tuning 3D human pose estimation model on the Human 3.6M dataset. The 3D human pose estimation results were also evaluated and compared with the baseline methods.
- We combined and integrated the proposed framework into a practical application for computing the angle of deviation of human poses in 3D space. This was applied for assessment and scoring in artistic gymnastics and training and dance assessment. Moreover, it operates in real-time on a PC with a low configuration.

The paper is organized as follows. Section 1 introduces human detection, 2D keypoint estimation, and 3D human pose estimation in the image and the applications. Section 2 discusses related works of the methods, the results of 2D keypoint estimation and 3D human pose estimation, and applications. Section 3 presents a combination of YOLOv5, context constraints, HR, TCM, and semi-supervised training methods for 3D keypoint estimation/3D human pose estimation. Section 4 introduces and presents the Human 3.6M dataset, evaluation metrics, implementations, results, discussions on 2D keypoint estimation, and 3D human pose estimation. Section 5 presents the application of computing the deviated angles on the 3D human skeleton. Section 6 concludes the paper and proposes some future work.

## 2. Related Works

Estimating human posture in 2D and 3D is of great research interest in computer vision. The results are applicable in many fields, especially in sports. In this paper, we are interested in estimating the human pose problem in 2D and 3D. The human pose estimation in 2D space is the human pose estimation in the color image obtained from monocular RGB images and videos. Three-dimensional human pose estimation determines the position of joints on the human skeleton, with each joint having coordinates (x,y,z).

**Figure 3:**
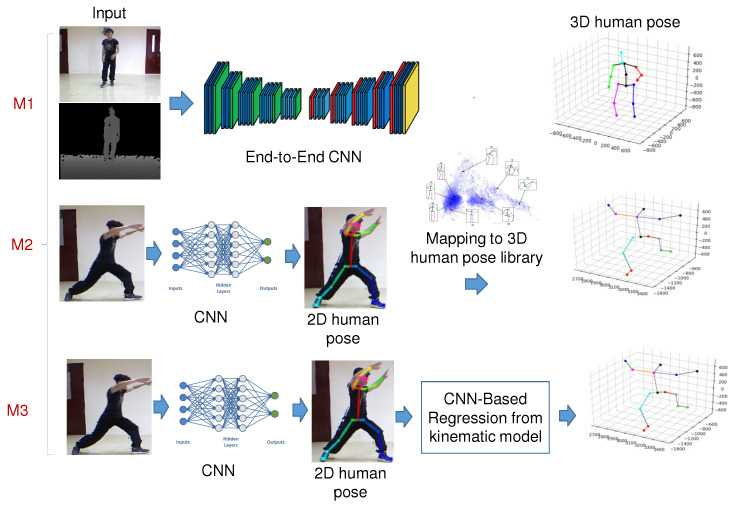
Illustrating three methods to estimate 3D human pose that use CNN-based from monocular RGB images/video.

Estimating the 2D human pose of a single person can be divided into direct regression methods and heat-map-based methods [22,28]. Direct regression methods are the end-to-end use of a CNN to learn a mapping from the input image to estimate the joints/2D keypoints or parameters of human skeleton models. The heat-map-based methods predict the locations of body parts and joints/2D keypoints from the heat map probability [28]. In addition, the two survey studies [22,28] detailed the results of 2D human pose estimation from a single-view camera. Two-dimensional multi-person pose estimation is performed by top-down methods or bottom-up methods. The top-down methods detect and classify each human in the image, constrain them by bounding boxes, then estimate the pose of each detected person. The bottom-up method includes two main steps: extracting local features by predicting skeleton joint candidates and skeleton joint candidate assembling for individual bodies. All four methods of 2D human pose estimation are illustrated in Figure 3 and 4 of [28].

In this paper, we present seven outstanding studies on estimating 2D human pose from RGB images or videos. Toshev et al. [16] proposed CNN-based regression (DeepPose) to regress the skeleton joints/2D keypoints. DeepPose uses a cascade of such regressors to refine the pose estimates and obtain better estimates from the estimated candidates. DeepPose includes seven layers (five convolutional layers and two fully connected layers), as shown in Figure 2 of [16]. DeepPose’s best results on the percentage of correct parts (PCP) at 0.5 on LSP are 61%. Tompson et al. [9] proposed a new CNN architecture with multi-resolution that uses a sliding window detector to produce a coarse heat map output. The model includes the heat-map-based parts model for coarse localization, a module to obtain and crop the convolution features at the (x,y) location for each joint/keypoint prediction, and fine-tuning model prediction. The loss function used in training is the mean-squared error (MSE) distance. The best results on PCKh@0.5 of the MPII dataset [29] are 82% with all joints of the human pose. Wei et al. [10] proposed convolutional pose machines (CPM); this CNN is a multi-stage architecture to be end-to-end trained for predicting joints/2D keypoints on heat maps. Stage 1 computes image features, and Stage 2 and up make the actual prediction based on the heat maps. The result of a previous stage is the predictive input for the next stage. The best results on PCKh@0.5 of the MPII dataset are 87.95%, and on the ankle (the most challenging part), results on PCKh@0.5 are 78.28%. The best result on PCKh@0.5 of LSP is 84.32%. Carreira et al. [11] proposed a feedforward architecture called iterative error feedback (IEF). This architecture can learn rich representations from the hierarchical feature extractors of both input and output spaces, by using the top-down feedback strategy. That is, after each training step, the error value of the feature set will be the feedback. The input of each layer is $x_{t}=I+g\left(y_{t}-1\right)$, where *I* is the image and $y_{t}-1$ is the output of the previous layer. The best results on PCKh@0.5 of the MPII dataset are 81.3%. Newell et al. [14] proposed a CNN called stacked hourglass network (SHN). The model consists of several hourglass (HG) modules arranged in series. Each HG processes input information from high to low resolutions and then from low to high resolutions. Thus, a single HG is a sort of full convolutional network. Such stacked HGs are for the improved inference across scales.

This scheme takes advantage of the characteristics and relationships of the human body parts. The low resolution will learn the position of the joints of the limbs; the higher resolution will learn the position of the limb and the relationship between the parts. The estimated result of the SHN network is much higher than that of the previously proposed networks, the average results on PCKh@0.5 of the MPII dataset being 90.9%. Xiao et al. [13] proposed a simple and effective strategy, called simple baselines (SB) for 2D human pose estimation and tracking. This network is a combination of a ResNet and several transposed convolution layers. The HG network uses upscaling (low resolutions to high resolutions) to increase the feature map resolution and set the convolutional parameters in the next blocks. The SB forms skip connections for each resolution. The mean results on the .mAP of the COCO dataset are 73.7% with ResNet-152, and the input size is 384×288. Sun et al. [12] proposed the high-resolution network (HR) for predicting the 2D keypoints/joints of the human body. Unlike SHN, HR performs prediction based on a high resolution to low resolution to high resolution representation in parallel and connects the multiple resolutions. HR does not perform any heat map supervision. The mean results on the .mAP of the COCO dataset are 77.0%.

Three-dimensional human pose estimation is usually performed based on two approaches [30]: the first is using DL networks, and the second is using the transformers (TranS) method.

**Figure 4:**
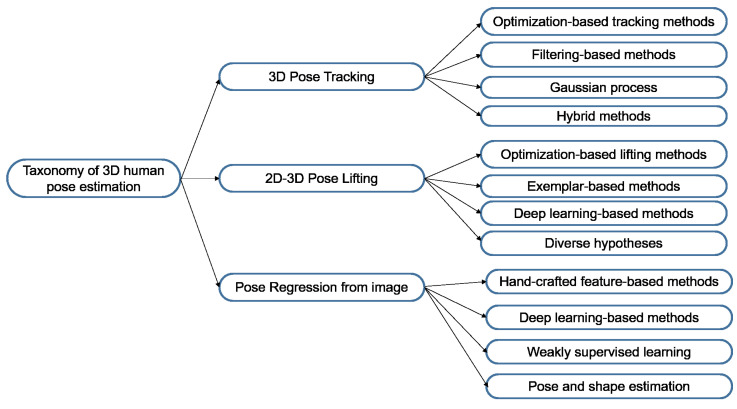
The taxonomy of 3D human pose estimation methods.

Regarding methods based on DL, estimating the 3D human pose of a person from monocular RGB images/videos can be performed based on three methods [22], illustrated in Figure 3: the first is using the CNNs end-to-end to estimate the 3D human pose (M1 in Figure 3); the second is to use the CNNs to lift the 2D human pose to the 3D human pose (M2 in Figure 3); the third is to use the CNN to regress the 3D human pose from the 2D human pose (M3 in Figure 3). The taxonomy of 3D human pose estimation is shown in Figure 4.

The results of 3D human pose estimation based on the two methods DL and TranS on the 3D human pose annotation of Human 3.6M is shown in Table 1.

The tree-dimensional HPE category has also received much research attention in the past decade. Wang et al. [20] conducted a full survey of 3D human pose estimation approaches, evaluation datasets, metrics, results, and applications. In this paper, we are only interested in 3D human pose estimation studies from monocular RGB images and videos. According to Song et al.’s study [57], the problem of the 3D human pose from monocular RGB images and videos generally is solved by two families of methods: direct 3D human pose estimation and 2D to 3D human pose lifting. However, the paper of Wang et al. [20] solved the problem of estimating 3D human pose from monocular RGB images and videos by three methods: direct 3D human pose estimation, 2D to 3D human pose lifting methods, and SMPL-based methods. Direct 3D human pose estimation is performed by designing an end-to-end CNN to predict the 3D coordinates of the joints of the 3D human pose from the images. This method includes two classes: detection-based methods and regression-based methods. Here, we introduce some typical studies for 3D human pose estimation. Pavlakos et al. [42] proposed a CNN for the end-to-end learning paradigm, including two works: a convolutional network (ConvNet) to predict the 2D joint location and a subsequent optimization step to recover the 3D coordinate joints of the 3D human pose. The mean per joint error (MPJE) (mm) on the Human 3.6M dataset was 51.9 mm and on the HumanEva-I dataset was 24.3 mm. Chen et al. [58] proposed a method based on using a CNN for 2D human pose estimation and 2D human pose matching with a 3D human pose library. The MPJE on the Human 3.6M dataset (protocol #1) was 69.05 mm.

In the category of the transformer methods, Zheng et al. [51] recently proposed the PoseFormer method. The authors designed a spatial–temporal transformer structure to follow the 3D pose of the person and then modeled the human pose and the relationship between joints within a frame and between frames. This method has the lowest average estimation error ever: the MPJE on the Human 3.6M dataset was 44.3 mm (Protocol #1) and 34.6 mm (Protocol #2). The best 3D human pose estimation rate was 320 fps with the input 2D human pose detected on a single GeForce GTX 2080 Ti GPU. Although the estimation accuracy is very high in the 3D human pose estimation process, this approach only focuses on estimating 3D human posture, but does not pay attention to the accuracy and processing time of the whole 3D human pose estimation process.

The applications of human pose estimation include some areas such as activity recognition, motion capture and augmented reality, training robots, and motion tracking for consoles [3,57]. Stenum et al. [1] developed an application that evaluates human body performance over the lifespan based on human pose estimation. At the same time, the authors also analyzed the challenges and limitations of human-posture-based applications as the problems of hidden body parts, limited training data, limited capture errors, limited positional errors, and limited recording devices. Badiola et al. [59] surveyed the number of studies on posture estimation and its applications. This provides an overview of this area of research in computer vision.

## 3. The Unified End-to-End YOLOv5-HR-TCM Framework

In papers [18,27,37,43,49,60,61], particular emphasis was placed solely on improving the 2D to 3D lifting process, and the 2D keypoint estimation process only uses 2D keypoint detectors such as ResNet, Mask RCNN, SHN, etc. Our study is interested in the results of all of the steps of the 3D human pose estimation process. We present the steps as follows.

### 3.1. Human Detection

Detecting humans in images using CNN has been studied extensively and has achieved impressive results. Many CNNs such as R-FCN [62], Faster RCNN [63], SSD [64], YOLO [65,66,67,68], and Faster RCNN [63] are presented and compared in Jonathan’s study [69]. An interesting model is Faster RCNN, which is an improvement of Fast RCNN [70]; it also integrates the region recommendation algorithm into the CNN model. Faster RCNN is based on two main ideas: building a single model consisting of a region proposal network (RPN) and Fast RCNN with a shared CNN. Inheriting Faster RCNN, He et al. [71] introduced the Mask RCNN based on Faster RCNN as the backbone for detecting and segmenting people in images. It achieves high accuracy, but the processing speed of Mask RCNN is relatively slow. To meet the requirement of fast computational time, YOLO appeared. YOLO is a CNN network with an average accuracy and very fast processing speed, up to 91 fps. Since the input is the input image, YOLO uses some simple steps of network convolution, pooling, and fully connected layers to obtain the output. This architecture can be optimized to run on a GPU with a single forward pass, and thus achieves very high speeds. The main idea of YOLOv1 [65] is to divide the image into a grid cell with a size (7×7). For each grid cell, the model will make predictions for a bounding box (B) of humans. Each box *B* includes five parameters (the coordinates of the center of the human (x,y), width (w) of the human, the height of the human (h), and the confidence $\left(cof_{h}\right)$ of the human prediction. Given the grid cells in the other (7×7) grid, the model also predicts the probability of each class of people. Confidence $cof_{h}$ is defined by Equation (1):

(1) $$cof_{h}=P\left(h\right)*IOU_{ground-truth}^{prediction}$$

where P(h) is the probability that there is a human in the ce and $IOU_{ground-truth}^{prediction}$ is the intersection over union of the prediction region and the ground truth.

YOLOv1 [65] imposes spatial constraints on bounding boxes: each grid cell can predict only very few bounding boxes and only one class. During training, the loss function does not have a separate evaluation between the error of the small bounding box versus the error of the large bounding box.

To improve the disadvantages of YOLOv2, YOLOv2 and YOLO 9000 have come up with some strategies: batch normalization, using the anchor box architecture to make predictions, direct location prediction, adding fine-grained features, multi-scale training, and a light-weight backbone. YOLOv3 [67] has a similar architecture to YOLOv2, but it also brings some improvements: using logistic regression to predict the confidence of the bounding box; using Darknet-53 as the backbone; using the feature pyramid network (FPN) architecture to make predictions from various scales of feature maps; adding associations between prediction classes.

The object detection challenge is now more accessible to those who do not have powerful computer resources thanks to the architecture of YOLOv4 [68]. Using YOLOv4, we can train an object detection network with extremely high accuracy using only a 1080ti or 2080ti GPU. To bring computer vision applications into practice in the future, current networks will need to be re-optimized to tolerate weak computing resources or develop high parallelism on servers.

In this paper, we use a pre-trained model trained on the COCO dataset of YOLOv5 [26] for head and human detection in a crowd and a context constraint to obtain the bounding box of the detected human in the image. When using YOLOv5 to detect people in the image of the Human 3.6M dataset, many other objects are mistakenly detected as persons. In the image of the Human 3.6M dataset, the person has the largest bounding box in the image. Therefore, we propose that the bounding box of the person is the bounding box with the highest height among the bounding boxes detected and marked as the person.

We compared the proposed method with some studies on human detection (e.g. Mask RCNN, VGG, SSD, Mobilenet) in images combined with constraints (**CC**). The results are shown in Table 2.

People detection results are near 100%, and the processing time is 55 fps on our PC. This is a very impressive result; the output of this step is the bounding box of the person detected in the image.

### 3.2. Two-Dimensional Human Pose and 2D Keypoint Estimation

**Figure 5:**
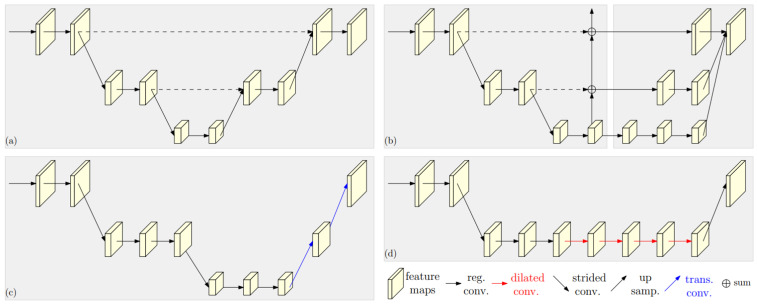
Illustration of the high-to-low and low-to-high processes [12] for 2D human pose and keypoint estimation of [13,14,79].

For human pose estimation and 2D keypoint estimation of people, one can use backbones such as ResNet [76], stacked hourglass networks (SHNs) [14], or some studies such as Openpose [77], 2D pose estimation using part affinity fields [78], convolutional pose machines (CPM) [10], cascaded pyramid network (CPN) [79], Simple Baselines [13], or DeeperCut [80]. The high-to-low and low-to-high frameworks performed with CNNs are stacked hourglass networks [14] (Figure 5a), cascaded pyramid network (CPN) [79] (Figure 5b), simple baselines [13] (Figure 5c), and DeeperCut [80] (Figure 5d), respectively, for estimating the human pose in the image.

**Figure 6:**
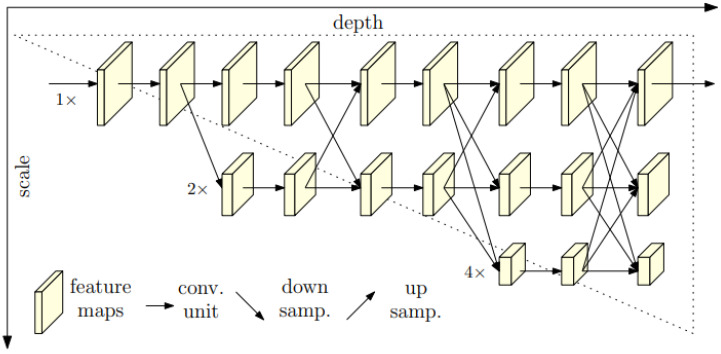
Illustration of HR architecture [12].

Figure 5 also shows that the high-to-low process of CNNs is sequential. While HR is presented in [12], it comes from the fact that when high-to-low convolutions are connected, the classification results at the region-level and pixel-level are low because this leads to enrichment of low-resolution representations, which means deterioration of high-resolution representations. HR implements parallel connections at the high-to-low resolution convolutions, which continuously strengthen multi-scale fusions across parallel convolutions of high-resolution representations, as illustrated in Figure 6. In particular, HR does not perform intermediate heat map supervision. Therefore, the accuracy of the keypoint detection and the computation time of HR is better than previous CNNs.

The aims of HR is to locate keypoints of the human pose in the image based on heat maps; the training model estimation process is the process of determining the value of the mean-squared error between the predicted heat maps and the ground-truth heat maps. The high-to-low network of HR includes four stages ($HR_{sr}$; s=1⋯4 is the stage number; r=1⋯4 is the resolution at the $s^{th}$ stage, its resolution is $\frac{1}{2^{r}-1}$ of the resolution of the first subnetwork), and the parallel processing of the subnetworks is shown as follows:

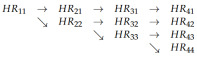



HR performs exchanging the information across the parallel multi-resolution subnetworks by repeating multi-scale fusions, as illustrated in Figure 3 and Formula 3 of [12].

The results of the accuracy of the 2D human pose/2D keypoint estimation on the COCO and MPII datasets are shown in Table 3 and Table 4, respectively. HR’s results are the most accurate.

Based on the results presented in Table 1 of the paper by Li et al. [18], the 2D keypoint estimation results are very good, from 4.4 to 5.4 pixels on cropped human images using HR. In this paper, we propose the method of using a person detector in the image and then using the person detection results for 2D keypoint/2D pose estimation, as illustrated in Figure 2. Our approach, called YOLOv5 + HR Combined, combines the pre-trained human detection model of YOLOv5 on the CrowdHuman dataset and HR.

### 3.3. Three-Dimensional Human Pose Estimation from Estimated 2D Human Poses

As presented in the works of Chen et al. [28] and Zheng et al. [86], single-person 3D HPE is based on two main methods: using the CNNs to estimate directly from the images and using the CNNs to estimate from 2D human pose/2D keypoint data (2D to 3D lifting). We performed a small survey on 3D human posture estimation methods in the Human 3.6M database, and the statistical results are in Table 1. Currently, the results of the transformer (TranS) models show that the 2D to 3D lifting method obtains results better than the results of CNNs models on the Human 3.6M dataset, as shown in Table 1 of [27] and Table 1. Therefore, we chose the approach of using the TranS method for estimating 3D human pose.

**Figure 7:**
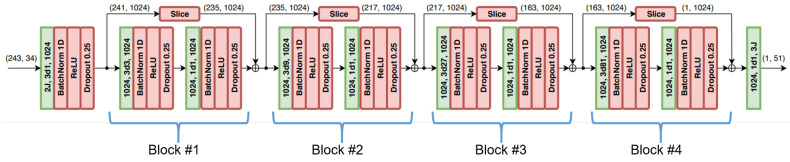
Illustration of the TCM architecture [27].

Pavllo et al. [27] proposed the temporal convolutional model (TCM) with the input as a 2D keypoint sequence. The input layer uses a 2D human pose of each frame and applies it to a temporal convolution with kernel size W=3, the output channels C=1024, and a dropout rate p=0.25; the number of blocks is 4; the tensor sizes are (243, 34); 243 frames is the receptive field and 34 channels (each frame is 17×2; 2 is the (x,y) dimensions), as illustrated in Figure 7.

In particular, the authors also proposed a semi-supervised training method by leveraging the unlabeled video for extending the supervised loss function with a back-projection loss term. There are two processes performed on the unlabeled video: the encoder implements 3D pose estimation from 2D joint coordinates, and the decoder is the back-projection of the estimated 3D pose to 2D joint coordinates.

## 4. Experimental Results

### 4.1. Data Collection, Implementations, and Evaluations

**Figure 8:**
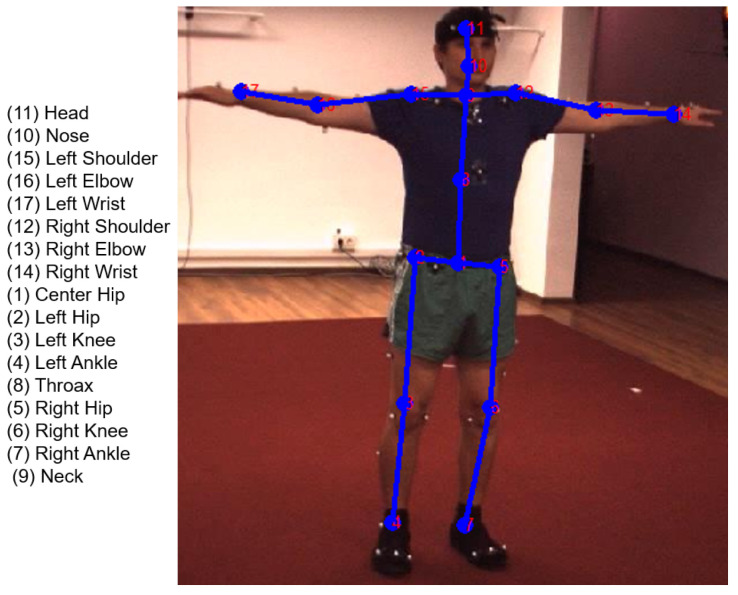
Example of 2D human pose/skeleton from the Human 3.6M dataset [19].

We used the benchmark Human 3.6M dataset [19] for evaluating the 2D human pose estimation/3D human pose estimation. Human3.6M is captured from 11 subjects/people (6 males and 5 females) in the Lab scene, which includes 16 daily activities (directions, discussion, greeting, posing, purchases, taking photos, waiting, walking, walking dog, walking pair, eating, phone talk, sitting, smoking, sitting down, miscellaneous). The frames were captured from time-of-flight (TOF) cameras, and the frame rate is from 25 to 50 Hz. Three-dimensional human pose annotations were marked by the MoCap system, and each pose includes 17 keypoints, as illustrated in Figure 8. In each human action, the camera’s intrinsic parameters are provided.

To evaluate the 2D human pose estimation, we used the camera’s intrinsic parameter set to define the 2D human pose annotation on the image. The 2D human pose annotations are projected from 3D human pose annotation by Equation (2):

(2) $$\begin{array}{cc} P2D.x & =\frac{P3D_{c}.x*fx}{P3D_{c}.z}+cx\\ P2D.y & =\frac{P3D_{c}.y*fy}{P3D_{c}.z}+cy \end{array}$$

where P2D is the coordinate of the keypoint in the image. $P3D_{c}$ is the coordinate of the keypoint in the camera coordinate system, which is computed by (3) [87].

(3) $$\begin{array}{cc} P3D_{c}.x & =\frac{\left(x_{d}-cx\right)*D\left(x_{d},y_{d}\right)}{fx}\\ P3D_{c}.y & =\frac{\left(y_{d}-cy\right)*D\left(x_{d},y_{d}\right)}{fy}\\ P3D_{c}.z & =depth(x_{d},y_{d}) \end{array}$$

where fx,fy,cx, and cy are the intrinsic parameters of the camera. Before converting from 3D to 2D, the coordinates of the $P3D_{c}$ joints in the camera coordinate system need to be determined based on Formula (4).

(4) $$P3D_{c}=\left(P3D_{w}-T\right)*R^{-1}$$

where *R* and *T* are the rotation and translation parameters to transform from the real-world coordinate system to the camera coordinate system. $P3D_{w}$ is the coordinate of the keypoint in the world coordinate system.

The training and testing data of the Human 3.6M dataset include three protocols: Pro #1 includes Subject #1, Subject #5, Subject #6, and Subject #7 for training and Subject #9 and Subject #11 for testing; Pro #2 is similar to Pro #1, but the predictions are further post-processed by a rigid transformation before comparing to the ground-truth; Pro #3 includes Subject #1, Subject #5, Subject #6, Subject #7, and Subject #9 for training and Subject #11 for testing.

In this paper, we used a PC with a Core I5 with GPU GTX 970, 4GB for fine-tuning, training, and testing 2D human pose estimation/3D human pose estimation. The programs were performed in the Python language (≥3.6 version) with the support of the CUDA 11.2/cuDNN 8.1.0 libraries. In addition, there are a number of other libraries such as Numpy, Scipy, Pillow, Cython, Matplotlib, Scikit-image, Tensorflow ≥ 1.3.0, etc.

For 2D human pose estimation evaluation, we evaluated the average 2D keypoint localization errors (A2DLEs) of 2D keypoints/2D human pose annotation ($P_{g}$) and the estimated 2D keypoints/2D human pose ($P_{e}$) in pixels. This is defined as the Euclidean distance between the 2D keypoints annotation and the estimated 2D keypoints, as in Equation (5).

(5) $$A2DLE=\frac{1}{N_{ac}}\sum_{1}^{N_{ac}}\frac{1}{N_{f}}\sum_{1}^{N_{f}}\frac{1}{17}\sum_{1}^{17}{\left(P_{e}-P_{g}\right)}^{2}$$

where $N_{ac}$ is the number of human actions, $N_{f}$ is the number of frames in the human action, and 17 is the number of keypoints of the human pose.

For 3D human pose estimation evaluation, we used the mean per joint position error (MPJPE) measurement, which is the mean Euclidean distance between estimated 3D joint positions ($P3D_{e}$) and 3D joint positions’ annotation ($P3D_{g}$), following Equation (6).

(6) $$MPJPE=\frac{1}{17}\sum_{1}^{17}{\left(P3D_{e}-P3D_{g}\right)}^{2}$$

The details of the protocols are as follows: Protocol (Pro) #1 uses the MPJPE measurement in millimeters (mm) to evaluate as [88]. Pro #2 uses the P-MPJPE (mm) [88,89]. Pro #3 uses the N-MPJPE [31] measurement.

### 4.2. Results and Discussions

The results of 2D keypoint estimation on Pro #1 of the Human 3.6M dataset are shown in Table 5. The results were evaluated on HR and its improved version, called Higher HR. The widths (w) of the high-resolution subnetworks in the last three stages were 32 (w32) or 48 (w48). The input image was resized to a fixed size (256 × 192)/(256×192) or (384 × 288)/(384 × 288) or (512 × 512)/(512) or (640 × 640)/(640). In Table 5, the error of the HR+ U + S [18] methods is the highest, A2DLE = 4.4 pixels. The HR+ U + S [18] and CPN [79] methods perform 2D keypoint estimation on the bounding box ground-truth of the human on the image. The results of the person detection are presented in Table 2. The results of the human detection step in the proposed method have an accuracy of close to 100%. The output of this step will be the input for 2D human pose estimation. The result in the next step of the proposed method (YOLOv5 + CC_HR_384_288) has an error of A2DLE = 5.14 pixels; this is the result of the estimated 2D keypoints on the bounding box of the YOLOv5 + CC detector. This result is better than the CPN+HR method [79] for human detection and 2D human pose estimation (A2DLE = 5.4). This shows that our proposed method for human detection is better with CPN [79].

The result of the method we proposed is very good; it is fully automatic with the input of the original image (1000×1002). The results of HR (HR_w48_384_288 [12], HR_w32_384_288 [12], HR_w32_256_192 [12], HR_w32_256_256 [12]) and Higher HR (Higher_HR_w48_640 [90], Higher_HR_w32_640 [90], Higher_HR_w32_512 [90]) have a high error, and we used the pre-trained model that was fine-tuned on the COCO dataset. The processing time of human detection and 2D keypoint estimation was 3.15 fps.

The results of 3D keypoint estimation/3D human pose estimation on Pro #1, Pro #2, and Pro #3 of the Human 3.6M dataset are shown in Table 6. In Table 6, we compare the proposed method with the 3D human pose estimation methods that have the best results currently. At the same time, we also present information about boxes (bounding box of human detection) and 2D keypoint estimation (the method used to estimate 2D human poses). The method that we propose has an accuracy equivalent to the 3D human pose estimation methods based on the human bounding box data, which is the ground-truth. The error on the MPJPE, P-MPJPE, and N-MPJPE measures of our proposed method on Pro #1, Pro #2, and Pro #3 is 46.5 mm, 37.0 mm, and 46.4 mm, respectively. The method we propose is much more accurate than the VNect (ResNet-50) [7] method (the error is 80.2 mm of Pro #1). In particular, our proposed method is slightly better (MPJPE = 50.5 mm) than the GraFormer [55,56] methods (MPJPE = 58.7 mm [55] and MPJPE = 51.8 mm [56]) for estimating 3D human pose. The GraFormer [55,56] method is a method based on a recent proposal.

In this paper, we also compare the processing time of the proposed method with the VNect [7] method on the Human 3.6M dataset when performed on a computer with a low configuration, as presented in Table 7.

**Figure 9:**
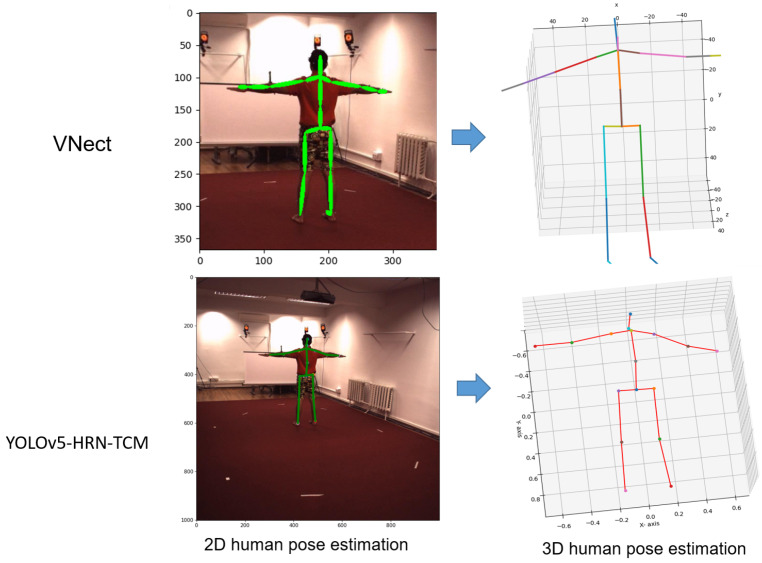
Illustrating of 2D and 3D human posture estimation. The VNect [7] method performs 2D human pose estimation on the resized image of (386×386) pixels. Our method proposes to estimate the human pose on a full-sized image (1000×1002 pixels).

The results of 2D human pose estimation and 3D human pose estimation are illustrated in Figure 9.

## 5. Pose-Based Application

**Figure 10:**
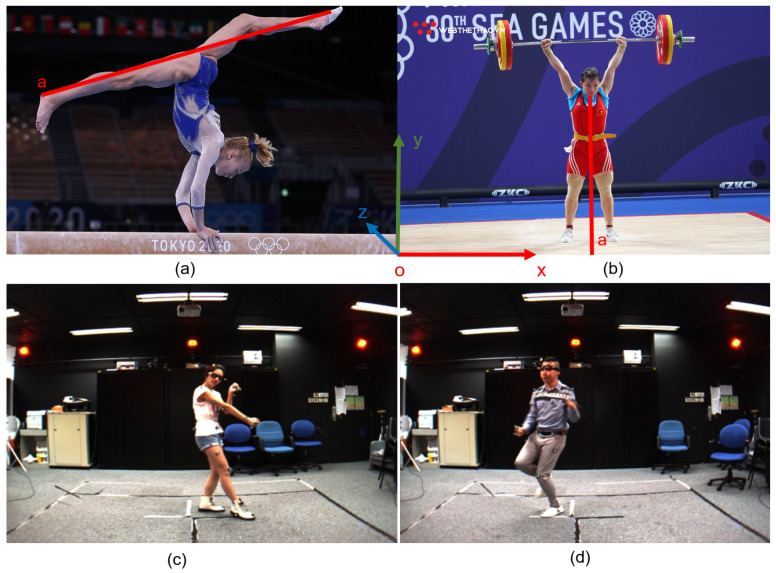
Illustration of computing movement scores in women’s artistic gymnastics (**a**) and weightlifting (**b**). Images (**c**) and (**d**) are an illustration of a dancer performing jazz and hip hop dances [95,96].

Figure 1 presents several applications based on human posture estimation. There have been studies using human posture to build applications in sports [59,92] and preserving and developing traditional martial arts [93,94]. Moreover, there is Zhang et al. [95], who published a dataset of human postures in martial arts, dancing, and sports. Scoring in sports competitions and martial arts performances have traditionally been based on the experts on the jury. The movements and actions of athletes are often very fast, so mistakes are inevitable. In particular, the assessment is based on the subjectivity and experience of the jury members. Therefore, having a system to support the process of assessing the accuracy of movements in sports competitions and martial arts performances has very high practical significance, as illustrated in Figure 10. Sports and martial arts competitions often take place in a larger space, so it is not reasonable to evaluate and score points based on the absolute coordinates of the person, bones, and joints. Therefore, we propose a rating and scoring system based on the deviation angle of the original shelf and important bones. Figure 10a,b illustrates calculating the angle $\left(\hat{\vec{a},\vec{ox}}\right)$ between the straight line of two legs with the ox axis; the smaller this angle, the higher the score is.

**Figure 11:**
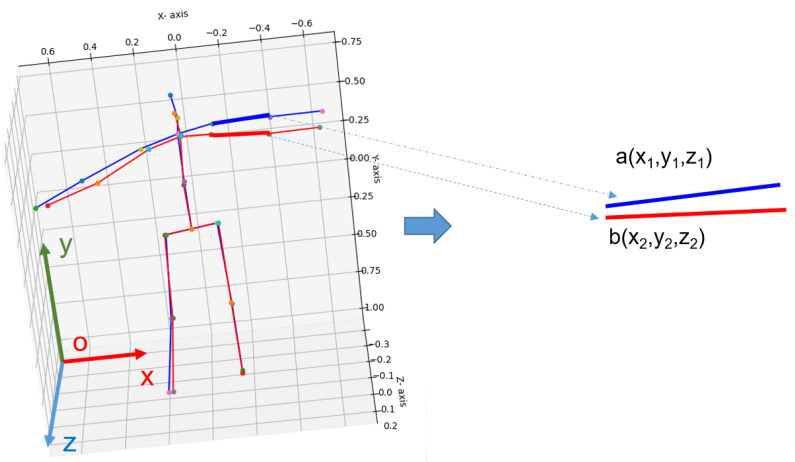
Illustration of the angle between a pair if bones between the estimated human skeleton and the ground-truth human skeleton in 3D space of the Human 3.6M dataset [19]. The left is the estimated human skeleton (red color) and the ground-truth human skeleton (blue color) in 3D space. The right shows the calculation of the angle between a pair of elbow bones.

In this paper, we propose an application based on the estimated human posture in 3D space. Our application is based on calculating the angle $A_{d}$ of a pair of bones between the estimated human skeleton and the ground-truth of the human skeleton, as illustrated in Figure 11. The deviation angle $A_{d}$ (as Equation (7)) is then averaged over the pair of bones $A\_av_{d}$ (as Equation (8)).

(7) $$\begin{array}{cc} A_{d}\left(\vec{a},\vec{b}\right) & =arccos\left(\vec{a},\vec{b}\right)=arccos\left(\frac{a.b}{|\vec{a}\left|\right|\vec{b}|}\right)\\ = & arccos(\frac{x_{1}y_{1}z_{1}+x_{2}y_{2}z_{2}}{\sqrt{x_{1}^{2}+y_{1}^{2}+z_{1}^{2}}\sqrt{x_{2}^{2}+y_{2}^{2}+z_{2}^{2}}}) \end{array}$$

where vector $\vec{a}$ has coordinates $\left(x_{1},y_{1},z_{1}\right)$ and vector $\vec{b}$ has coordinates $\left(x_{2},y_{2},z_{2}\right)$.

(8) $$A\_av_{d}=\frac{1}{16}\sum_{1}^{16}A_{d}$$

Based on the assessment and scoring of women’s artistic gymnastics [97,98,99], as illustrated in Figure 10a, we propose how to evaluate and score the “Execution Score: execution, artistry, composition and technique” contest, as shown in Table 8. In Table 8, if the angle of deviation is 2 degrees, subtract 0.1 points.

As illustrated in Figure 10c,d, the human skeleton in dance teaching (hip hop, jazz) by experts and coaches is the original source of data for teaching and assessing the accuracy of movements. In this paper, we propose a method of assessment and scoring in dance teaching based on the deviation angle between the experts’ human skeleton (ground-truth) and the estimated human skeleton of the trainees. The details of the assessment and scoring are shown in Table 9. In Table 9, if the angle of deviation is 1 degree, subtract one point.

**Figure 12:**
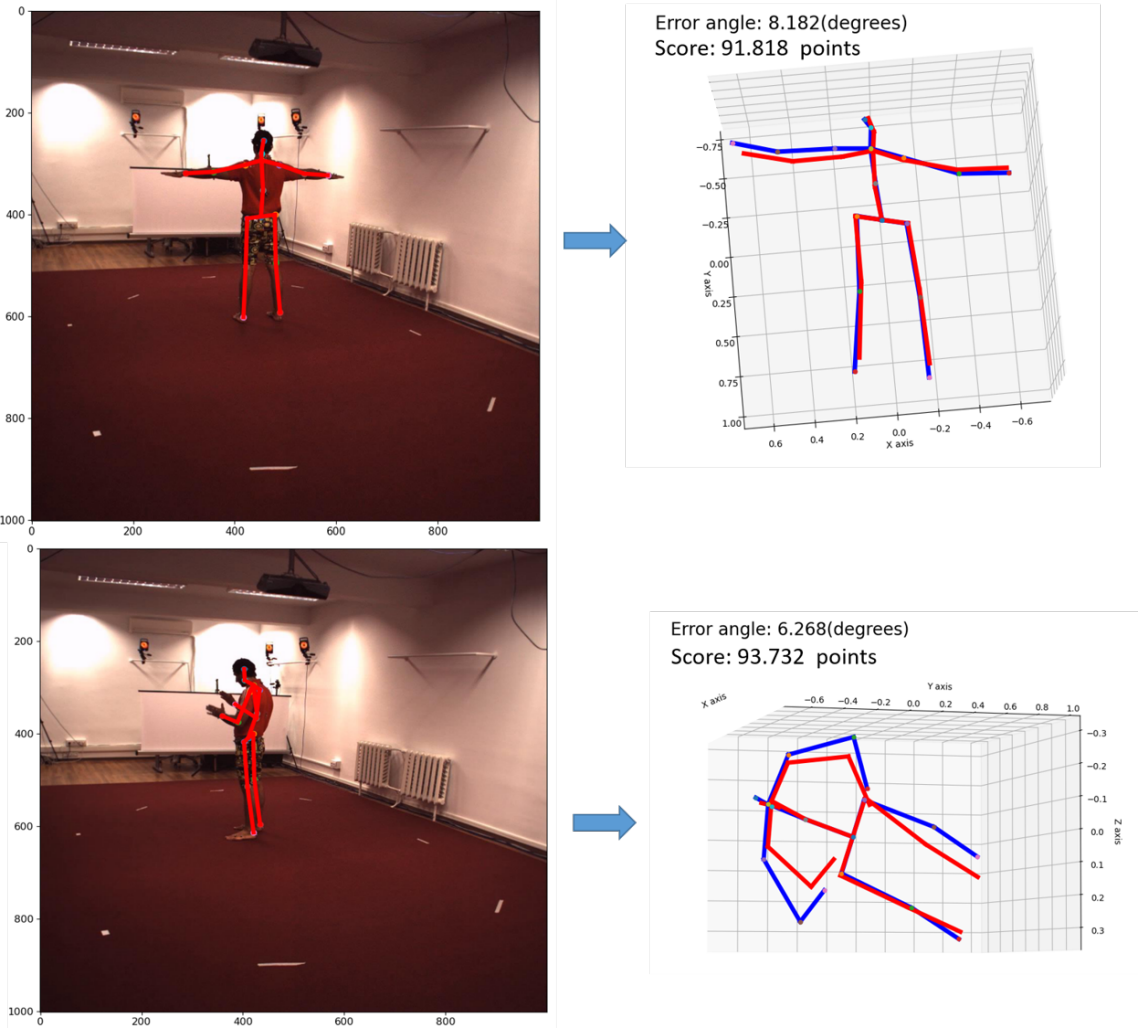
Illustration of the human posture estimation in color images (**left**) and the result of human posture estimation in 3D space (**right**). The blue skeleton is the ground-truth; the red skeleton is the estimated skeleton of the proposed YOLOv5-HR-TCM framework.

The results of the deflection angle between the pairs of bones on the Human 3.6M dataset are shown in Table 10. The average deviation angle between the estimated 3D human skeleton bones and the 3D human skeleton bones’ ground-truth is 8.2 degrees; the scoring results based on Table 9 are illustrated in Figure 12. If the calculation is based on the average deviation angle of Table 9, the scoring system gives 100−8.2=91.8 points.

The worst-case scenario is when the estimated angle error is 90 degrees, so the error rate of the current application is $\frac{8.2}{90}*100=9.11\%$. This is a relatively large error, but is calculated on average over 16 human bones. However, in practical applications in sports, we are often only interested in some bones in the human body. As shown in Figure 10a, we are only interested in the angle between the legs and the shelf; the smaller the angle, the higher the score is. Figure 10b shows the results when we are interested in the angle between the “Thorax-Neck” and the ground floor; the closer the angle is to 90 degrees, the higher the score is.

**Figure 13:**
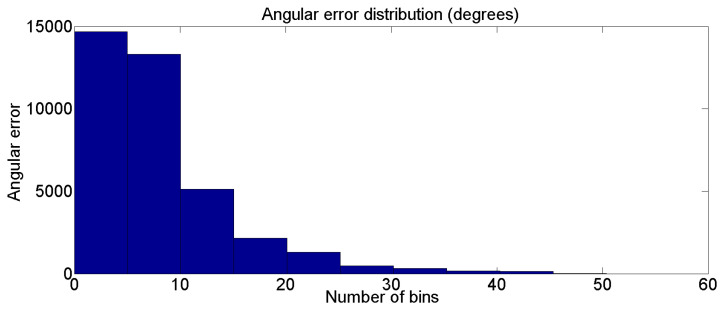
Deviation angle distribution between estimated 3D human skeleton bones and 3D human skeleton ground-truth on the dataset (s_09_act_02_subact_01_ca_01) of Human 3.6M dataset.

Based on the angular results in Table 10, we show the distribution of the deviation angle of the set “s_09_act_02_subact_01_ca_01” of the Human 3.6M dataset in Figure 13. Error distribution results are all concentrated in the range from 0 to 10 degrees.

Figure 12 shows the estimated results of 2D human pose and 3D human pose. Scores based on the rating in Table 9 are also shown.

The entire source code of the sports scoring and estimation system is stored in link ( https://drive.google.com/drive/folders/1WRr-L3IcH_lhSqMUJDaw1v23OBRdTXPC?usp=sharing, (accessed on 12 May 2022)).

Thus, our proposed method can perform end-to-end 3D human pose estimation at a rate of 3.146 fps, which can be improved on computers with higher frames for real-time speed response in a gym fitness center. However, the proposed model also has some limitations that currently only estimate the pose of a human in the image. Therefore, it usually applies only to certain non-competitive sports, but only to performance and scoring sports such as skating, gymnastics, weightlifting, etc.

## 6. Conclusions and Future Works

Estimation of 2D human pose and 3D human pose has been studied extensively in recent years. However, studies often focus on improving the accuracy of the estimation results. In terms of 2D human pose estimation and 3D human pose estimation processing time, especially in building applications based on 3D human pose estimation, there are still many limitations. This paper accomplished two main tasks: (1) We proposed a unified end-to-end framework for estimating the 3D human pose from color image input data, named YOLOv5-HR-TCM. The proposed framework is a combination of current best approaches at each step of the estimation process, such as human detection of color images, estimating the human pose on the bounding box of the detected human, and estimating the quantity of 3D human pose from 2D human pose (2D to 3D lifting method). (2) An application was built for assessment and scoring in artistic gymnastics, sports competitions, and assessment of teaching dance, traditional martial arts, and sports. In the near future, the survey and evaluation of the combination at each step such as human detection, 2D human posture estimation (e.g., EfficientHRet [100], YOLO-POSE [101]), and 3D human posture estimation (e.g., GraFormer [56]), to choose the best method in each step to build the best overall model, will be performed. Specifically, we will apply the results of 3D human posture estimation to many sports applications, human activity recognition, and sports analysis. More specifically, the evaluation test of the 3D human posture estimation model is based on color images, and scoring is based on the angle of deviation of the weightlifter in the gym fitness center.

## Figures and Tables

**Table 1:** Statistics of the result of studies based on the MPJPE(mm) measurement on the ground-truth of the Human3.6M dataset for 3D human pose estimation.

Method	Model	Results of Mean per Joint Position Error (MPJPE) (mm)
Rhodin et al. [31]	DL	Protocol #1: 131.7
Tome et al. [32]	DL	Protocol #1: 88.39; Protocol #2: 70.4 Protocol #3: 79.6
Mehta et al. [7]	DL	ResNet 100: 82.5; ResNet 50: 80.5
Zhou et al. [6]	DL	Protocol #1: 64.9
Wang et al. [33]	DL	Protocol #1: 63.67
Veges et al. [34]	DL	Protocol #1: 61.1
Fang et al. [35]	DL	Protocol #1: 60.4; Protocol #2: 45.7 Protocol #3: 72.8
Omran et al. [36]	DL	Protocol #1: 59.9
Zhao et al. [37]	DL	Protocol #1: 57.6
Nibali et al. [38]	DL	Protocol #1: 57.0; Protocol #2: 40.4
Moon et al. [39]	DL	Protocol #1: 53.3; Protocol #2: 34.0
Lee et al. [40]	DL	Protocol #1: 52.8; Protocol #2: 43.4
Li and Lee et al. [41]	DL	Protocol #1: 52.7; Protocol #2: 42.6
Pavlakos et al. [42]	DL	Protocol #1: 51.9
Kocabas et al. [43]	DL	Protocol #1: 51.83
Bastian et al. [44]	DL	Protocol #1: 50.9
Tekin et al. [45]	DL	Protocol #1: 50.12
Karim et al. [46]	DL	Protocol 1: 49.9
Sun et al. [47]	DL	Protocol #1: 49.6
Rhodin et al. [48]	TranS	Protocol #1: 46.8
Chen et al. [24]	DL	Protocol #1: 46.3; Protocol #2: 37.7 Protocol #3: 50.3
Martinez et al. [49]	DL	Protocol #1: 45.5
Li et al. [50]	TranS	Protocol #1: 43.7; Protocol #2: 35.2
Zheng et al. [51]	TranS	Protocol #1: 44.3; Protocol #2: 34.6
Hossain et al. [52]	DL	Protocol #1: 39.2
Wang et al. [53]	DL	Protocol#1: 37.6
Pavllo et al. [27]	TranS	Protocol #1: 37.2; Protocol #2: 27.2
Pavllo et al. [54]	DL	Protocol #2: 36.0
Zhao et al. [55]	TranS	Protocol #1: 35.2
Zhao et al. [56]	TranS	Protocol #1: 35.2

**Table 2:** The results of human detection on the Human 3.6M dataset (Pro #1) evaluated on the CNNs.

Measurement/Methods	Number Testing Samples	Number Detected	${\mathrm{AP}}_{50}$ (%)	${\mathrm{AP}}_{55}$ (%)	${\mathrm{AP}}_{60}$ (%)	${\mathrm{AP}}_{65}$ (%)	${\mathrm{AP}}_{70}$ (%)	Processing Time (fps)
YOLOv5 [26] + CC	548,819	548,346 (99.91%)	99.78	99.38	98.42	97.07	94.16	55
Mask RCNN [71,72] + CC	548,819	548,819 (100%)	97.17	96.93	96.61	96.12	95.51	2
MobilenetV1 SSD [73,74] + CC	548,819	507,991 (92.56%)	96.87	95.66	93.59	89.52	81.38	10
VGG SSD [74] + CC	548,819	536,496 (97.75%)	99.14	98.60	97.66	95.99	92.81	12
Mobilenet SSD [75] + CC	548,819	548,801 (99.99%)	77.04	75.93	73.43	68.34	59.99	4.34

**Table 3:** Results of single-person keypoint detection [12] on the test set of the COCO dataset.

Method	Backbone-Size of Input	OSK (Object Keypoint Similarity) (%)		
${\mathrm{AP}}^{50}$	${\mathrm{AP}}^{75}$	AP
OpenPose [77]	VGG-19	84.9	67.5	61.8
Mask-RCNN [71]	Faster RCNN	87.3	68.7	63.1
CPN [79]	ResNet-Inception—384 × 288	91.4	80.0	72.1
Simple Baseline [13]	ResNet-152—384 × 288	91.9	81.1	73.7
HR-W32 [12]	HR-W32—384 × 288	92.5	82.8	74.9
HR-W48 [12]	HR-W48—384 × 288	92.5	83.3	75.5
HR-W48 + extra data [12]	HR-W48—384 × 288	92.7	84.5	77.0

**Table 4:** Results of single-person keypoint detection [12] on the test set of the MPII datasets.

Method	Average of PCK@0.5(%)
DeeperCut [80]	88.5
SHNs [14]	90.9
Hourglass Residual Units (HRUs) [81]	91.5
Generative Adversarial Net.-SHNs [82]	91.8
Adversarial PoseNet [58]	91.9
Pyramid Residual Module (PRMs) [83]	92.0
Multi-scale structure-aware CNN [84]	92.1
Stacked U-Nets [85]	92.3
SimpleBaseline [13]	91.5
HR-W32 [12]	92.3

**Table 5:** The 2D keypoint estimation/2D human pose estimation results on Pro #1 of the Human 3.6M dataset.

Method	Number of Parameters (Million(M))	Input Size of Image	Average Joint Localization Error (A2DLE) (pixels)	Processing Time (fps)
HR_w48_384_288 [12]	63.6M	Full size (1000 × 1002)	52.4	3.144
HR_w32_384_288 [12]	28.5M	Full size (1000 × 1002)	54.3	3.14
HR_w32_256_192 [12]	28.5M	Full size (1000 × 1002)	58.2	3.145
HR_w32_256_256 [12]	28.5M	Full size (1000 × 1002)	56.7	3.14
CPN [79]	-	Bounding-box of detected person	5.4	-
HR+ U + S [18]	63.6M	Bounding-box of detected person	4.4	-
Higher_HR_w48_640 [90]	63.6M	Full size (1000 × 1002)	40.0	2.5
Higher_HR_w32_640 [90]	28.6M	Full size (1000 × 1002)	40.2	2.6
Higher_HR_w32_512 [90]	28.6M	Full size (1000 × 1002)	40.8	2.88
Ours(YOLOv5+CC_HR_384_288)	63.6M	Full size (1000 × 1002)	5.14	3.15

**Table 6:** Illustration of 3D keypoint estimation/3D human pose estimation on Pro #1, Pro #2, and Pro #3 of the Human 3.6M dataset.

Method	2D Keypoints Estimation	BBoxes	Blocks	Receptive Field (frames)/ N. Epochs	MPJPE (mm) (Pro #1)	P-MPJPE (mm) (Pro #2)	N-MPJPE (mm) (Pro #3)
TCM + semi-sup. [27]	HR_w48_384_288	HR_w48_384_288	4	243/80	216.2	184.4	215.8
TCM + semi-sup. [27]	HR_w32_384_288	HR_w32_384_288	4	243/80	216.3	184.2	215.9
TCM + semi-sup. [27]	HR_w32_256_192	HR_w32_256_192	4	243/80	217.2	186.3	217.1
TCM + semi-sup. [27]	HR_w32_256_256	HR_w32_256_256	4	243/80	216.4	185.8	216.0
TCM + semi-sup. [27]	HR_w48_384_288	Ground-truth	4	243/80	124.6	104.3	123.8
TCM + semi-sup. [27]	HR_w32_384_288	Ground-truth	4	243/80	125.4	107.9	124.5
TCM + semi-sup. [27]	HR_w32_256_192	Ground-truth	4	243/80	124.22	105.7	123.8
TCM + semi-sup. [27]	HR_w32_256_256	Ground-truth	4	243/80	123.7	105.3	123.5
TCM + semi-sup. [27]	CPN	Mask RCNN	4	243/80	46.8	36.5	-
TCM + semi-sup. [27]	CPN	Ground-truth	4	243/80	47.1	36.8	-
TCM + semi-sup. [27]	CPN	Ground-truth	3	81	47.7	37.2	
TCM + semi-sup. [27]	CPN	Ground-truth	2	27	48.8	38.0	
TCM + semi-sup. [27]	Mask RCNN	Mask RCNN	4	243/80	51.6	40.3	
TCM + semi-sup. [27]	Ground-truth	Ground-truth	4	243/80	37.2	27.2	35.4
3d-pose-baseline [49]	SHN	SHN (cro. 440 × 440)	-	-/200	62.9	47.7	-
Cas.(Full-sup.) [18]	HR_w32_384_288	HR_w32_384_288	3	-/200	49.7	37.7	-
Adversarial Lea. [60]	SHN	SHN	-	-/90	58.6	37.7	-
SemGCN [37]	SHN	SHN	-	-/200	57.6	-	-
CVAE-based [61]	SHN + 2DPoseNet	SHN + 2DPoseNet	-	-/200	58.0	40.9	-
RootNet+PoseNet [39]	Mask RCNN	Mask RCNN	-	-/20	54.4	-	-
Multi-View Sup. [31]	ResNet-50	ResNet-50	-	-/-	-	64.6	-
Pose SS (PSS) [43]	ResNet-50	ResNet-50	-	-/140	65.3	57.2	-
Cas. (Weakly-sup.) [18]	HR_w32_384_288	-	2	-/200	60.8	46.2	-
VNect (ResNet-50) [7]	ResNet-50	ResNet-50	-	-/-	80.5	-	-
Vnect (ResNet-100) [7]	ResNet-100	ResNet-100	-	-/-	82.5	-	-
Boosting [91]	SHN	SHN	-	-/2	88.8	66.5	-
GraFormer [55]	SHN	SHN	-	-/100	58.7	-	-
GraFormer [55]	Ground-truth	Ground-truth	-	-/100	35.2	-	-
GraFormer [56]	SHN	SHN	-	-/100	51.8	-	-
GraFormer [56]	Ground-truth	Ground-truth	-	-/100	35.2	-	-
Our	YOLOv5_HR _384_288	YOLOv5_HR _384_288	4	243/80	50.5	37.0	45.7
Our	YOLOv5_HR _384_288	Ground-truth	4	243/80	46.5	35.0	44.4

**Table 7:** Processing time of 3D human pose estimation on the Human 3.6M dataset.

Method	Processing Time (FPS)
VNect [7]	1.36
Our (YOLOv5-HR-TCM)	3.146

**Table 8:** The method of evaluating and scoring women’s artistic gymnastics with the contest “Execution Score”.

Error Angle (Degrees)	Score (Points)
0	10
2	9.9
4	9.8
...	...

**Table 9:** The method of evaluating and scoring dance training.

Error Angle (Degrees)	Score (Points)
0	100
1	99
2	98
...	...

**Table 10:** Deviation angle (A_Av_d - degrees) between estimated 3D human skeleton bones and 3D human skeleton ground-truth based on the Human 3.6M dataset (Pro #1).

Bone Pairs	Mean Deviation Angle (A_Av_d) (Degrees)
Center Hip-Right Hip	6.2
Right Hip-Right Knee	6.0
Right Knee-Right Ankle	6.9
Center Hip-Left Hip	6.2
Left Hip-Left Knee	5.1
Left Knee-Left Ankle	7.9
Center Hip-Thorax	6.8
Thorax-Neck	6.0
Neck-Nose	14.2
Nose -Head	10.1
Neck-Left Shoulder	9.0
Left Shoulder-Left Elbow	8.7
Left Elbow-Left Wrist	9.9
Neck-Right Shoulder	9.6
Right Shoulder-Right Elbow	8.8
Right Elbow-Right Wrist	10.1
Average	8.2

## Data Availability

Not applicable.
